# Interpretation errors in focused cardiac ultrasound by novice pediatric emergency medicine fellow sonologists

**DOI:** 10.1186/s13089-018-0113-4

**Published:** 2018-12-09

**Authors:** Rosemary Thomas-Mohtat, Craig Sable, Kristen Breslin, Jacqueline G. Weinberg, Aparna Prasad, Lauren Zinns, Joanna S. Cohen

**Affiliations:** 1 Department of Emergency Medicine and Trauma Services, Children’s National Medical Center, Washington, DC USA; 2 Department of Cardiology, Children’s National Medical Center, Washington, DC USA; 3 Department of Cardiology, Children’s Hospital of Pittsburgh, Pittsburgh, PA USA; 4 Department of Cardiology, Goryeb Children’s Hospital, Morristown, NJ USA; 5 Department of Emergency Medicine, Icahn School of Medicine at Mt. Sinai, New York, NY USA; 6 Faculty, George Washington University School of Medicine, Washington, DC USA

**Keywords:** Point of care ultrasound, Focused cardiac ultrasound, Education, Pediatric emergency medicine

## Abstract

**Background:**

Focused cardiac ultrasound (FOCUS) is a core competency for pediatric emergency medicine (PEM) fellows. The objectives of this study were (1) to evaluate test characteristics of PEM-fellow-performed FOCUS for pericardial effusion and diminished cardiac function and (2) to assess image interpretation independent of image acquisition.

**Methods:**

PEM fellows performed and interpreted FOCUS on patients who also received cardiology service echocardiograms, the reference standard. Subsequently, eight different PEM fellows remotely interpreted a subset of the PEM-acquired and cardiology-acquired echocardiograms.

**Results:**

Eight PEM fellows performed 54 FOCUS exams, of which two had pericardial effusion and four had diminished function. PEM fellow FOCUS had a sensitivity of 50.0% (95% CI 9.19–90.8) and specificity of 100.0% (95% CI 91.1–100.0) for detecting diminished function, and sensitivity of 50.0% (95% CI 2.67–97.33) and specificity of 98.1% (95% CI 88.42–99.9) for detecting pericardial effusions. When PEM fellows remotely interpreted 15 echocardiograms, the sensitivity was 81.3% (95% CI 70.7–88.8) and specificity 75% (95% CI 67.0–81.0) for detecting diminished function, and sensitivity of 76.3% (95% CI 65.0–85.0) and specificity 94.4% (95% CI 89.0–97.0) for detecting pericardial effusion. There were no differences in sensitivity and specificity of PEM fellows’ interpretation of FOCUS studies compared to their interpretation of cardiology echocardiograms. Interrater reliability for interpretation of remote images (kappa) was 0.66 (95% CI 0.59–0.73) for effusion and 0.31 (95% CI 0.24–0.38) for function among the fellows.

**Conclusion:**

Novice PEM fellow sonologists (a physician who performs and interprets ultrasound) in the majority of instances were able to acquire and remotely interpret FOCUS images with limited training. However, they made real-time interpretation errors and likely need further training to incorporate real-time image acquisition and interpretation into their practice.

**Electronic supplementary material:**

The online version of this article (10.1186/s13089-018-0113-4) contains supplementary material, which is available to authorized users.

## Background

In 2010, the American Society of Echocardiography and the American College of Emergency Physicians released a consensus statement on the importance of focused cardiac ultrasound (FOCUS) on patient care and treatment in an emergency setting [1]. Subsequently, the American Academy of Pediatrics and the American College of Emergency Physicians have released policy statements stressing the importance of training for pediatric emergency medicine (PEM) fellows in point of care ultrasound (POCUS). They recommend a structured POCUS curriculum and competency assessment for PEM fellows [2]. An expert panel of PEM physicians trained in POCUS, included FOCUS as one of the core ultrasound exams for this curriculum [3].

PEM providers who have completed a dedicated extra year of POCUS fellowship training can diagnose pericardial effusion and abnormalities of function with high sensitivity and specificity [4–6]. However, there are limited data on competency assessment of PEM fellows with routine training in POCUS during their PEM fellowships. Our primary objective was to assess PEM fellows’ accuracy in assessing cardiac function and the presence of pericardial effusions while performing FOCUS exams with limited training. Our secondary objective was to assess the accuracy of PEM fellows’ interpretive skills of both FOCUS and complete echocardiograms in a remote classroom setting. We hypothesized that PEM fellows would perform better on interpretation of remotely acquired images than on images acquired in real time.

## Materials and methods

This study took place at a freestanding tertiary care children’s hospital with approximately 90,000 emergency department (ED) visits per year and with a cardiology service that performs 21,000 echocardiograms a year. PEM fellows completed FOCUS training, consisting of a 1 h lecture on echocardiography, a month of basic POCUS training with regular supervised ultrasound scans, and quality assurance sessions with PEM POCUS-trained faculty. In addition, each PEM fellow competed a minimum of 10 FOCUS exams reviewed by PEM POCUS-trained faculty. The fellows were taught to determine function by qualitatively evaluating how closely the anterior leaflet of the mitral value approached the interventricular septum in the parasternal long axis view. They were taught to evaluate pericardial effusion in all four views. FOCUS exams included four standard cardiac views: the parasternal long, parasternal short, apical 4 chamber, and subxiphoid views. Specifically in the parasternal long axis view, they were taught to note any anechoic structures above the descending aorta, within the pericardium.

A convenience sample of patients’ ages 1 month to 21 years, with an indication for an echocardiogram as determined by a pediatric cardiologist, were enrolled between April 2014 and June 2015. The patients were enrolled in the pediatric ED, cardiology clinic, or the inpatient service. The indications for the echocardiograms included chest pain, syncope, altered mental status, hypoxia, and follow-up of known cardiac disease. Premature infants with post-conception age less than 36 weeks and patients who were less than 8 day post-sternotomy were excluded. Because the cardiology echocardiogram was the reference standard, we also excluded patients if they experienced significant therapeutic interventions or changes in clinical conditions between the FOCUS exam and the cardiology echocardiogram. This included progression of illness requiring transfer of the patient to a higher level of care, need for emergent surgery, initiation of ECMO, intubation, or the initiation of intravenous vasopressor agents.

Eight PEM fellows performed FOCUS, using a Sonosite M-Turbo^**®**^ ultrasound system (Bothell, WA). Within 24 h of FOCUS, a cardiologist or echocardiography technician performed an echocardiogram with a Phillips IE 33 ultrasound system (Andover, MA). PEM fellows were blinded to the results of the cardiology echocardiogram and vice versa. The PEM fellows were blinded to the clinical status of the patient to the extent possible; the PEM fellow performing the FOCUS did not access the medical record or discuss the clinical case with the patient or with the provider caring for the patient. However, we could not blind the fact that a patient was in the resuscitation bay or the ICU, for example.

The PEM fellows performing FOCUS completed a case report form (CRF) immediately following the performance of the FOCUS. The CRF included the fellows’ interpretation of the presence or absence of significant pericardial effusion and the qualitative global systolic function as either normal or depressed. We used dichotomous qualitative variables for pericardial effusion (absent/trivial versus present) and cardiac function (normal versus depressed) rather than attempting to quantify amount of fluid or ejection fraction.

A board-certified pediatric cardiologist, blinded to the patient’s clinical presentation, chief complaint, cardiac history and PEM fellow interpretation of FOCUS, reviewed and interpreted the recorded clips of each FOCUS and cardiology echocardiogram. This cardiologist used a CRF with a similar format to the PEM fellows to subjectively note the presence or absence of significant effusion, the qualitative global function and if the quality of the study was adequate to perform these assessments. The blinded interpretation of the cardiology-performed echocardiogram was used as the reference standard for subsequent analysis.

In the second phase of the study, PEM fellow interpretation skills were assessed remotely in a classroom setting. Thirty matched studies were compiled: 15 FOCUS exams performed by PEM fellows and 15 cardiology-acquired echocardiograms on the same patients. We selected studies, so that 5 of these sets had pericardial effusions, 5 had diminished function, and 5 were normal. Eight PEM fellows were shown these studies in random order and were asked to comment on the presence or absence of pericardial effusion and global cardiac function. PEM fellows were not given any clinical information about the patients. Only the four standard views were presented. Only one of these eight PEM fellows had acquired images in the first phase of the study.

IBM SPSS version 24 (Armonk NY) and Microsoft Excel (Microsoft, Inc, Redmond, WA) were used to calculate test characteristics of PEM fellow FOCUS exams, with cardiology echocardiograms as the reference standard. Similar methodology was used to calculate test characteristics of PEM fellows’ interpretation of both FOCUS and cardiology studies in the remote setting.

## Results

Eight PEM fellows enrolled a convenience sample of 54 patients (Table 1).

**Table 1:** Demographics of recruited patients (*N* = 54)

Male	29 (54%)
Location	
ED	18 (33%)
Cardiology clinic	28 (52%)
Inpatient	4 (7%)
ICU	4 (7%)
Congenital heart disease, present	16 (30%)
Age in years [median (IQR)]	8 (1.75, 14.25)

Two (4%) patients had pericardial effusions and 4 (8%) had diminished function. The median time to complete the FOCUS was 6 min (IQR 5, 9). The median time difference between the two ultrasounds (PEM and cardiology) was 34 min (IQR 17.5, 58.8). No patients were excluded for changes in clinical status or interventions. The expert cardiologist did not find any PEM FOCUS studies with technical limitations precluding the determination of the two variables of interest.

### Phase 1

**Fig. 1:**
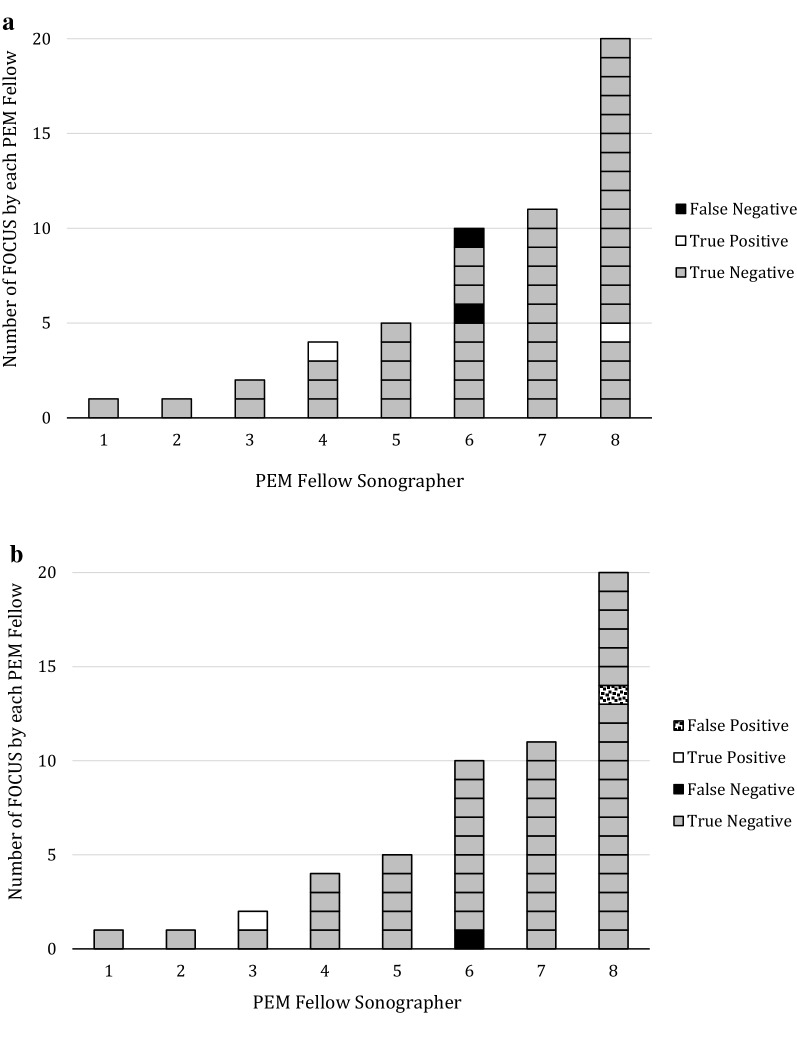
Test characteristics of FOCUS performed by PEM fellows

Results of the bedside FOCUS studies compared with the cardiology echocardiograms for our two variables of interest are shown in Tables 2 (diminished function) and 3 (pericardial effusion). The distributions of test characteristics of PEM fellow performed and interpreted FOCUS are shown in Fig. 1. Video clips of the four errors are available in Additional file 1: Phase S1, Phase S2, Phase S3, Phase S4.

**Table 2:** Results of FOCUS compared with cardiology echocardiograms for diminished function

	Cards+	Cards−
FOCUS+	2	0
FOCUS−	2	50

**Table 3:** Results of FOCUS compared with cardiology echocardiograms for pericardial effusion

	Cards+	Cards−
FOCUS+	1	1
FOCUS−	1	51

The sensitivity of PEM fellow performed and interpreted FOCUS was 50.0% (95% CI 9.19–90.8) with a specificity of 100.0% (95% CI 91.1–100.0) for detecting diminished function. The sensitivity was 50.0% (95% CI 2.67–97.33) and specificity of 98.1% (95% CI 88.42–99.9) for detecting pericardial effusions (Table 4).

**Table 4:** Performance metrics of real-time interpretation of FOCUS (Phase 1)

	Diminished function	Pericardial effusion
Sensitivity, % (95% CI)	50.0 (9.19–90.8)	50.0 (2.67–97.33)
Specificity, % (95% CI)	100.0 (91.1.0–100.0)	98.1 (88.42–99.9)

All three cases of missed pathology were by a single 2nd year fellow. The one missed pericardial effusion was in an inpatient with a structurally normal heart with a clinically significant effusion. One of the patients with missed diminished function was a clinic patient with a structurally normal heart with mild to moderately diminished left systolic function. The second patient with missed diminished function was an ED patient with known congenital heart disease and moderately decreased right and left systolic function.

### Phase 2

**Fig. 2:**
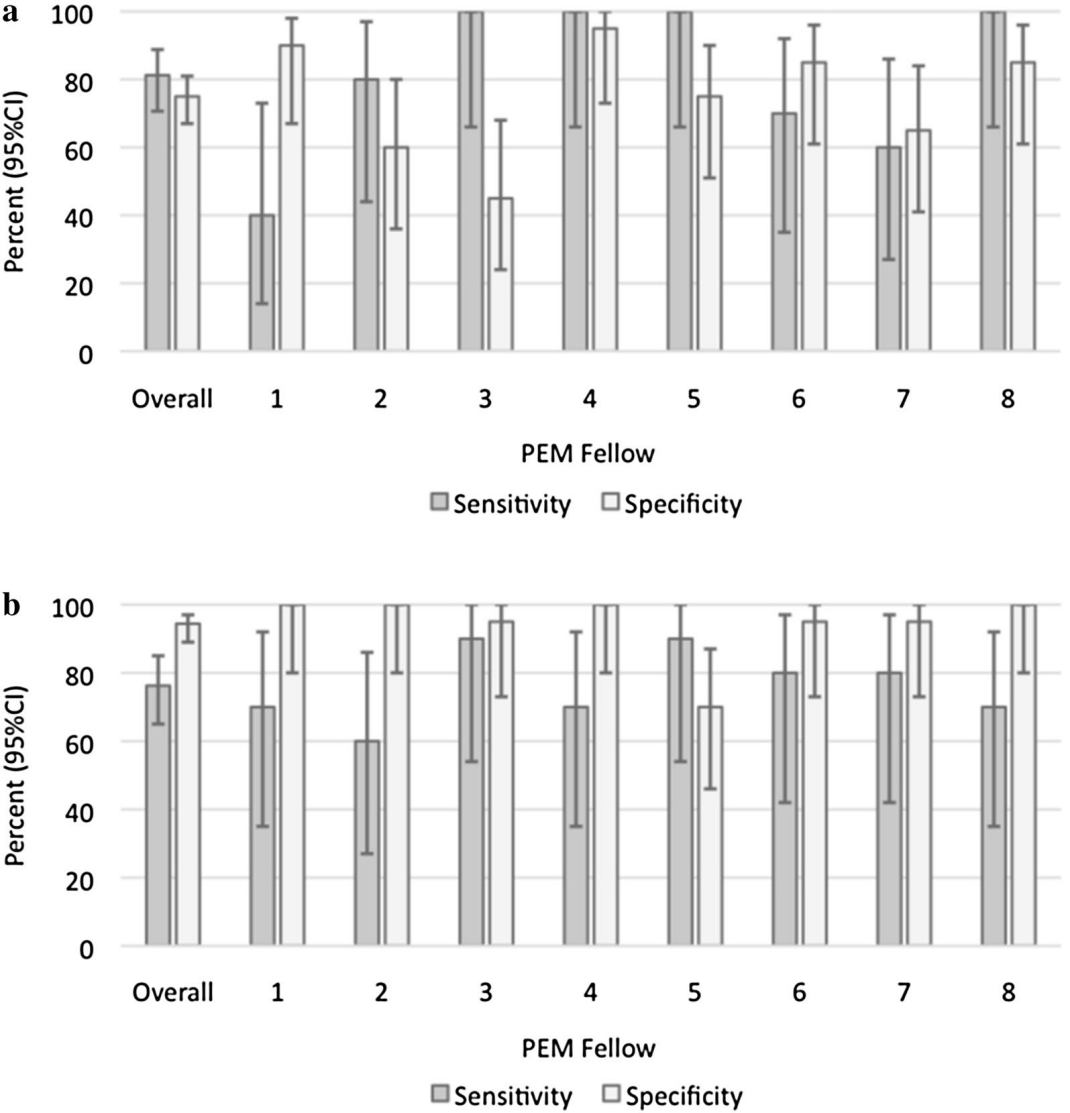
Classroom test of images (Phase 2). Overall sensitivity and specificity of interpretation of abnormal studies by 8 PEM fellows for function (**a**) and effusion (**b**)

The test characteristics of remote interpretation of 15 sets of images by a different set of similarly trained eight PEM fellows are shown in Table 5. The sensitivity was 81.3% (95% CI 70.7–88.8) and specificity was 75% (95% CI 67.0–81.0) for detecting diminished function. The sensitivity was 76.3% (95% CI 65.0–85.0) and specificity was 94.4% (95% CI 89.0–97.0) for detecting pericardial effusion. Individual performance of each fellow remotely is detailed in Fig. 2.

**Table 5:** Performance metrics of overall classroom interpretation of combined FOCUS and cardiology echocardiograms (Phase 2)

	Diminished function	Pericardial effusion
Sensitivity, % (95% CI)	81.3 (70.7–88.8)	76.3 (65.0–85.0)
Specificity, % (95% CI)	75.0 (67.0–81.0)	94.4 (89.0–97.0)

There was no statistical significant difference between the sensitivity of detecting diminished function (*p* = 0.13) or pericardial effusion (*p* = 0.48) during real-time interpretation and classroom interpretation. There was also no significant difference between specificity during real-time interpretation and remote interpretation for detecting effusion (*p* = 0.3). There was, however, a statistically significant improvement in specificity when interpreting function in real time (100%) as compared to the remote interpretation (75%) (*p* < 0.01).

We calculated the interobserver reliability of fellows’ interpretation of effusion and diminished function among each other in Phase 2. The kappa for effusion was 0.66 (95% CI 0.59–0.73) and the kappa for function was 0.31 (95% CI 0.24–0.38).

**Fig. 3:**
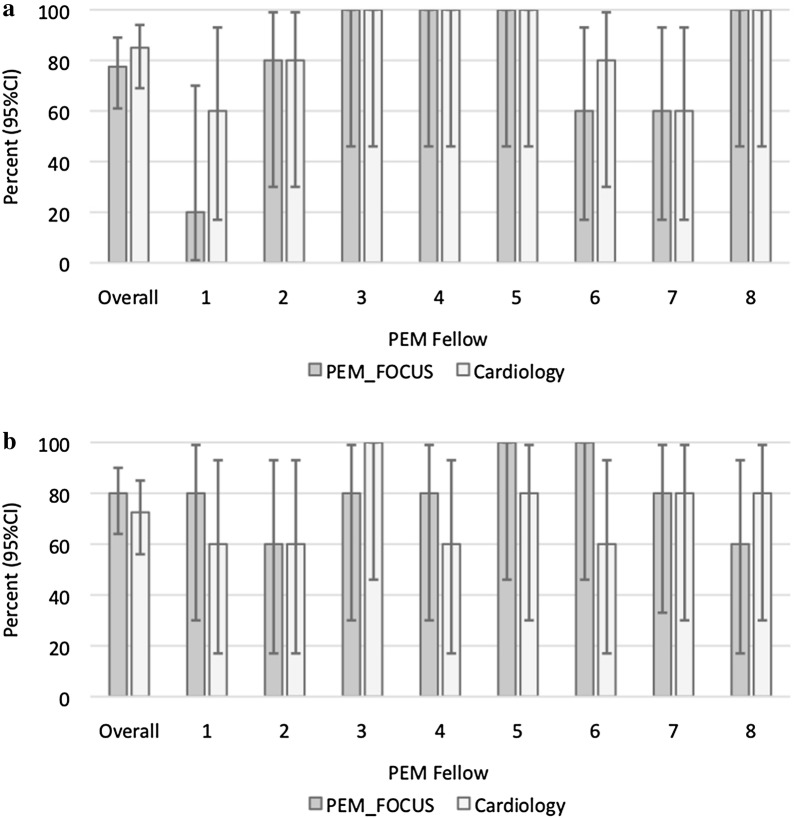
Sensitivity of interpretation of classroom test of images of PEM FOCUS versus cardiology images by 8 PEM fellows for function (**a**) and effusion (**b**)

There was no statistically significant difference in the sensitivity or specificity of detection of diminished function or pericardial effusion when comparing PEM fellows’ remote interpretations of FOCUS images to the same PEM fellows’ remote interpretations of cardiology-acquired echocardiograms (Tables 6 and 7). Performance metrics of classroom interpretation of individual fellows is presented in Figs. 3 (sensitivity) and 4 (specificity). Select videos of errors made in Phase 2 are included in Additional file 2: Phase S5, Phase S6, Phase S7, Phase S8.

**Table 6:** Performance metrics of classroom interpretation of FOCUS of diminished function (Phase 2)

	FOCUS	Cardiology echo	*p*
Sensitivity, % (95% CI)	77.5 (61.0–89.0)	85.0 (69.0–94.0)	0.57
Specificity, % (95% CI)	73.8 (63.0–83.0)	76.3 (65.0–85.0)	0.86

**Table 7:** Performance metrics of classroom interpretation of FOCUS of pericardial effusion (Phase 2)

	FOCUS	Cardiology echo	*p*
Sensitivity, % (95% CI)	80.0 (64.0–90.0)	72.5 (56.0–85.0)	0.60
Specificity, % (95% CI)	95.0 (87.0–98.0)	93.8 (85.0–98.0)	0.60

**Fig. 4:**
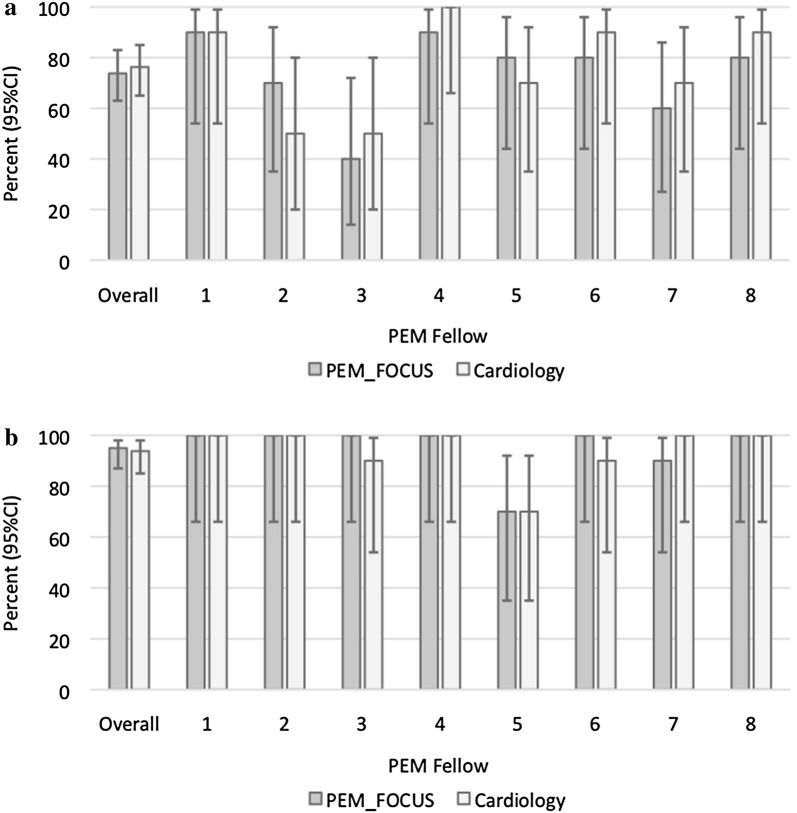
Specificity of interpretation of classroom test of images of PEM FOCUS versus cardiology images by 8 PEM fellows for function (**a**) and effusion (**b**)

## Discussion

This is the first study that has evaluated PEM fellow performance of FOCUS and specifically evaluated remote interpretation of FOCUS distinctly from acquisition. We demonstrated that with limited training, PEM fellows are able to acquire FOCUS images of sufficient quality to allow interpretation of the presence or absence of pericardial effusion and the presence or absence of significantly diminished cardiac function. The reviewing cardiologist found all FOCUS to be of sufficient technical quality to make these determinations. PEM fellows in our study had improved sensitivity when interpreting FOCUS images remotely in a classroom setting, suggesting that some fellows may face challenges of simultaneous acquisition and interpretation while at the bedside. When remotely evaluating echocardiograms, PEM fellows showed similar ability to interpret FOCUS exams and cardiology obtained exams, also suggesting that the deficit in interpreting the FOCUS exams, while obtaining them was less related to the quality of the image acquired. In addition, the interpretation of pericardial effusion had greater interobserver agreement among the fellows than determination of diminished function, as it is an easier assessment to make.

These findings suggest that novice PEM fellow sonographers may need more training in real-time interpretation while scanning at the bedside. Prior research showing more accurate interpretation of FOCUS has involved a larger number of training scans. Current recommendations from the American College of Physicians are for 25–50 scans per application, but the exact number is still debated [7]. Emergency medicine faculty with 10 h of mixed didactic and hands on scanning and more than 45 ultrasounds and adult non-cardiology residents with 12 h of mixed didactic and hands on scanning with a mean of 33 ultrasounds showed increased competency in FOCUS [8, 9]. Blehar et al. [10] evaluated learning curves of acquisition and interpretation of POCUS by emergency medicine residents; they found that acquisition of FOCUS had no plateau point on the learning curve (i.e., that it was consistently difficult to achieve mastery), and the interpretation learning curve did not plateau for FOCUS until the learners had performed about 30 scans.

In addition, we found that novice PEM fellow sonographers were able to more accurately interpret their own images remotely, suggesting that the fact of being at the bedside during interpretation added a layer of complexity. This may be due to perceived time or performance pressure at the bedside, which could be exacerbated in real-time resuscitation situations. For this reason, we think that real-time image interpretation is a critical skill for PEM fellow sonographers.

Given the low prevalence of pericardial effusion and depressed cardiac function in the pediatric patient population overall, other modes of encountering pathology such as via simulation or review of image banks may be necessary to improve interpretation by novice sonologists. Both low and high fidelity ultrasound simulation modalities are available [11]. A 1-day workshop using multi-faceted simulation based education and assessment of focused cardiac ultrasound in the evaluation of the hypotensive patient, showed improved competency in emergency medicine residents [12].

When PEM fellows performed and interpreted FOCUS at the bedside, the study had high specificity but poor sensitivity for identifying pericardial effusion and diminished cardiac function. Eliminating the data of the one fellow who had the 3 misses from the analysis would yield a sensitivity of 100% for both correct identification of pericardial effusion and diminished function by PEM fellows.

This study has several limitations. First, this was performed at a single institution. Results at other hospitals may be different. Second, there were few patients with cardiac disease. Therefore, the point estimates of sensitivity and specificity can be skewed by one or two incorrect readings, with the resultant wide confidence intervals for these estimates. Most of the incorrect interpretations were obtained by one fellow, which suggests a need for individual remediation rather than a more global problem. FOCUS image acquisition and interpretation may or may not be generalizable to other novice FOCUS learners, but reviewing errors or misclassification is an integral part of any quality assurance program. We did not study implementation of a FOCUS protocol in the emergency room [13].

In addition, by design and due to logistic issues, we did not study chamber size abnormalities, cardiac standstill, or determination of cardiac arrest. International recommendations have been made for the use of FOCUS in cardiac arrest; while feasibility of using FOCUS in cases of pediatric cardiac arrest has been published, adult studies have noted challenges in agreement of interpretation of cardiac standstill among different levels of providers [14–16].

## Conclusion

In this small study, PEM fellows for the most part were able to obtain ultrasound images of sufficient quality to allow interpretation of diminished cardiac function and pericardial effusion. However, interpretation was better performed remotely than at the bedside by PEM fellows. There was greater interrater agreement for pericardial effusion than diminished function by the novice PEM sonographer. Prior to completion of the current recommendation of 25 scans, novice PEM fellow sonologists are likely to make real-time acquisition and interpretation errors, and therefore, supervision and quality assurance are necessary. We recommend more extensive training in focused cardiac ultrasound. Future studies may wish to incorporate a severity-enriched patient population or use other modes of presenting pathology such as pediatric ultrasound image banks that are being developed.

## Additional files

**Additional file 1: Phase S1.** Missed diminished function (False Negative). Parasternal long axis view. Image acquisition made interpretation difficult. The gain setting is a bit dark to visualize the interventricular septum and the anterior leaflet of the mitral valve. The captured video would be improved by including the inferior portion of left ventricle and apex. **Phase S2**. Missed diminished function (False Negative). Parasternal long axis view. Image acquisition made interpretation difficult. Similar to Additional file Phase 1(a) gain setting is a bit dark and the left ventricle is not well imaged. **Phase S3**. Normal interpreted as effusion (False Positive). Subxiphoid view. Image acquisition made interpretation difficult, again with the gain setting too dark. Trace or trivial amount of pericardial effusion was considered negative for our study; it was listed as “None/ Trivial” on the data collection form. **Phase S4**. Missed pericardial effusion (False Negative). Parasternal long axis view. Image acquisition was adequate, while gain could be increased. This was a gross operator interpretation error.

**Additional file 2: Phase S5.** Missed pericardial effusion (False Negative). Parasternal long axis view. Image acquisition is good. This was an interpretation error. A pericardial effusion is seen above the descending thoracic aorta and pericardium. Also of note, there is also a small pleural effusion present, seen as the anechoic strip below the pericardium and lateral to the descending aorta. Phase S6. Missed pericardial effusion (False Negative). Parasternal long axis view. Small pericardial effusion is visible in this view. This was an interpretation error. Phase S7. Normal function called abnormal (False Positive). Parasternal long axis view. Images acquisition is acceptable. This was an interpretation problem. The anterior leaflet touches the interventricular septum during diastole with relatively normal chamber proportions. Phase S8. Normal function called abnormal (False Positive). Apical 4 chamber view. Image acquisition is adequate. Other views were also available that showed normal function.

## Abbreviations

FOCUS: focused cardiac ultrasound
PEM: pediatric emergency medicine
POCUS: point of care ultrasound
